# Supplementary material for: The role of genetic essentialism and genetics knowledge in support for eugenics and genetically modified foods
Source: PLoS One. 2021 Sep 30;16(9):e0257954. doi: 10.1371/journal.pone.0257954 (PMC8483317; doi:10.1371/journal.pone.0257954)
Supplement: S1 Appendix — (DOCX) [file pone.0257954.s001.docx]

**S1 Appendix**

**Genetic Essentialistic Tendencies Measure**

From Dar-Nimrod, I., Ruby, M. B., Cheung, B. Y., Tam, K.-P., & Murray, D. (2014). *The four horsemen of genetic essentialism: Theoretical underpinnings, methodological advancements, and empirical findings*. Symposium presented at the 2014 SPSP Annual Meeting, Austin, TX, USA.

All items were completed on a scale that ranged from 1 (Strongly Disagree) to 5 (Strongly Agree) in Study 1 and from 1 (Strongly Disagree) to 7 (Strongly Agree) in Study 2.

| 1. A person with a genetic predisposition for obesity is destined to be fat. |
| --- |
| 1. If someone has a genetic predisposition for cancer, sooner or later they will get cancer (unless they die at an early age). |
| 1. People with a genetic predisposition to a certain personality are destined to behave in a certain way. |
| 1. People with a genetic predisposition to be intelligent eventually will show intellectual achievements. |
| 1. People with a genetic predisposition for Alzheimer’s will eventually show signs of the memory loss (unless they die at an early age). |
| 1. A person with a genetic predisposition for creativity will always be creative. |
| 1. An individual's particular behavior is not changeable if it has genetic basis. |
| 1. Finding a gene that is associated with depression means that a therapy targeting that specific gene is likely the only way to cure a patient of depression. |
| 1. The environment does not affect the chances of getting cancer for someone with a genetic susceptibility to cancer. |
| 1. People without genetic predisposition for intelligence will be unintelligent even with intelligent parents and a stimulating learning environment. |
| 1. Even in an environment which encourages and nurtures creative behavior, a person without a genetic predisposition for creativity will still be uncreative. |
| 1. In the long run, the environment doesn’t affect the likelihood of a person becoming an alcoholic if that person has a genetic predisposition for alcoholism. |
| 1. Criminals who have a gene associated with criminality are more similar to each other than they are to criminals with no such gene. |
| 1. People with a genetic predisposition for alcoholism are more similar to each other than they are to people with no such predisposition. |
| 1. People who have a gene for insomnia are probably quite similar. |
| 1. People with a genetic predisposition for intelligence are more similar to each other than they are to people with no such predisposition. |
| 1. People with a gene associated with kindness are more similar to one another than those who do not have the gene. |
| 1. People with a gene associated with risk taking are probably quite similar. |
| 1. It is natural for a person with a genetic predisposition to athleticism to excel in athletics. |
| 1. It is natural for a person with a genetic predisposition for insomnia to sleep badly. |
| 1. It is more natural for a person with a genetic predisposition for criminality to be an outlaw than for a person without such a gene. |
| 1. Finding that there is a gene for risk taking makes this sort of behavior a natural thing. |
| 1. It is natural to behave aggressively if one has genetic predisposition to aggression. |
| 1. It is natural that a person with a genetic predisposition for creativity is considered to be a creative by other people. |
