# Supplementary material for: The role of genetic essentialism and genetics knowledge in support for eugenics and genetically modified foods
Source: PLoS One. 2021 Sep 30;16(9):e0257954. doi: 10.1371/journal.pone.0257954 (PMC8483317; doi:10.1371/journal.pone.0257954)
Supplement: S2 Appendix — (DOCX) [file pone.0257954.s002.docx]

**S2 Appendix**

**Eugenics Acceptance Measure**

All items were completed on a scale ranging from 1 = “strongly disagree” to 5 = “strongly agree” in Study 1 and a scale ranging from 1 = “strongly disagree” to 7 = “strongly agree” in Study 2.

Cronbach’s alpha = .93

| **Eugenics Item** | **Mean (SD) Study 1** | **Mean (SD) Study 2** |
| --- | --- | --- |
| We should change the tax laws to discourage poorer people from having so many kids. | 2.57 (1.22) | 3.02 (1.38) |
| The government should pay families to abort a fetus with genetic defects. | 1.95 (1.08) | 2.64 (1.42 |
| Less educated people are having too many kids. | 3.05 (1.25) | 3.37 (1.33) |
| Society would be better off in the long run if we could encourage highly educated people to have more children than less educated people. | 2.51 (1.14) | 3.06 (1.35) |
| Once a poor family already has two kids, the parents should be paid by the government to be sterilized. | 1.95 (1.11) | 2.63 (1.43) |
| Sterilization of those possessing undesirable traits (e.g., a disorder) is a way to improve future generations. | 2.04 (1.13) | 2.71 (1.40) |
| It will be to the detriment of society if violent offenders have biological children. | 2.45 (1.07) | 2.94 (1.32) |
| There should be more research into technology that allows for designer babies. | 2.10 (1.10) | 2.77 (1.42) |
| There should be laws discouraging people with low intelligence from having biological children. | 1.84 (1.00) | 2.68 (1.42) |
| There should be tax incentives for people with mental illnesses to prevent them from having biological children. | 2.05 (1.11) | 2.75 (1.37) |
| Reproduction should be reserved for people who do not have mental illnesses. | 1.98 (1.04) | 2.75 (1.42) |
| People with a criminal record should be prevented from having biological children. | 1.83 (0.93) | 2.63 (1.43) |
| People should need to have a reproduction license to have kids. | 2.00 (1.20) | 2.72 (1.47) |
| The government should require the sterilization of those who have serious genetic defects. | 1.90 (1.05) | 2.70 (1.44) |
| Anyone convicted of a violent crime should be sterilized as part of their punishment. | 1.98 (1.09) | 2.74 (1.42) |
