# Supplementary material for: The role of genetic essentialism and genetics knowledge in support for eugenics and genetically modified foods
Source: PLoS One. 2021 Sep 30;16(9):e0257954. doi: 10.1371/journal.pone.0257954 (PMC8483317; doi:10.1371/journal.pone.0257954)
Supplement: S3 Appendix — (DOCX) [file pone.0257954.s003.docx]

**S3 Appendix**

**Measure of Genetics Knowledge**

(items came from Christensen, Jayaratne, Roberts, Kardia, & Petty, 2010 and Jallinoja & Aro, 1999).

Correct response bolded.

1. When they’re born, identical twins have exactly the same genes. (**1=agree**, 2=disagree)
2. On average, a person has half their genes in common with their siblings. (**1=agree**, 2=disagree)
3. A mother and biological daughter who look alike have more genes in common than a mother and biological daughter who do not look alike. (1=agree, **2=disagree**)
4. There are different types of genes in different parts of the body. (1=agree, **2=disagree**)
5. Single genes directly control specific human behaviors. (1=agree, **2=disagree**)
6. Do plants that are not genetically modified still contain genes? (**1=yes**, 2=no)
7. What sex chromosomes does a man typically have? (1=XX, **2=XY**)
8. How many pairs of chromosomes do humans have? (text entry: 23)

For items 9-21, 1=correct, 2= not correct (correct response in brackets)

1. Healthy parents can have a child with a hereditary disease. (1)
2. The carrier of a disease gene may be completely healthy. (1)
3. All serious diseases are hereditary. (2)
4. The genotype is not susceptible to human intervention. (1)
5. Gene is a molecule that controls hereditary characteristics. (1)
6. The child of a disease gene carrier is always also a carrier of the same disease gene. (2)
7. A gene is a piece of DNA. (1)
8. Genes are inside cells. (1)
9. A gene is a cell. (2)
10. A gene is a part of a chromosome. (1)
11. Genes are bigger than chromosomes. (2)
12. Different body parts include different genes. (2)
13. It has been estimated that a person has about 20,000 to 25,000 genes. (1)
