# Supplementary material for: The role of genetic essentialism and genetics knowledge in support for eugenics and genetically modified foods
Source: PLoS One. 2021 Sep 30;16(9):e0257954. doi: 10.1371/journal.pone.0257954 (PMC8483317; doi:10.1371/journal.pone.0257954)
Supplement: Attachment — Submitted filename: Response to Reviewers R2.docx [file pone.0257954.s004.docx]

Dear Dr. Gao,

We are grateful for the opportunity to revise our manuscript and to address the critiques of the reviewers. Below, we describe our responses to each of the reviewers’ points in the order that they appear. Our responses are entered right beneath each of the reviewers’ specific comments.

Reviewer #1: Thank you for addressing my comments. Nice work!

There is a character limit for this box, so ignore this.

We thank the reviewer for their positive assessment.

Reviewer #2: Here are some comments on the current version of the manuscript.

I have one new comment on the GMO Attitudes Measure:
You write: “Participants completed the 9-item measure from Scott et al., (2016). Five of these items asked about support of GMO products (e.g., “Your government forbidding imports of genetically modified (GM) foods from other countries” and participants indicated their support on a 9-point scale from 1 (Certainly oppose) to 9 (Certainly support). Four of these items asked about perceived risk of GMO products (e.g., Genetically modified foods having unknown side-effects, increasing risks of cancer or other diseases for people who consume them” which participants answered on a 9-point scale from 1 (No risk at all) to 9 (Extreme risk). The latter subscale was reverse coded so that higher scores meant more favorable attitudes. This scale has been used in previous research investigating the bases of people’s support and opposition to GMO foods (e.g., Royzman, Cusimano, & Leeman, 2017). Cronbach’s α = 0.93. "
In the first example item, indicating 9 = certainly support means you support forbidding imports of GMO foods, meaning you oppose GMO foods. You say you only reverse score the risk items and then average all the items together. Is that true? If so, that’s a major issue, because you are averaging together 5 (not reverse-scored) items where high scores indicate opposition and 4 (reverse-scored) items where high scores indicate support. I’m pretty sure it’s just a mistake in the writeup, looking at your code and the data. If it is a mistake in the writeup, please confirm that all items were reverse coded and rewrite this methods paragraph. If this was done on purpose, please explain more thoroughly why.

We thank the Reviewer for catching this mistake. We did in fact reverse-code all items, not just the risk items, so that higher scores indicate more support for GMO foods. We have now corrected this on page 14 where we now say “Both subscales were reverse coded so that higher scores meant more favorable attitudes towards GMO foods.”

Also, while looking at the data file/code to try to understand this, I noticed you only have 3 gmo risk items in your data file, not four as stated in the methods section.

We thank the Reviewer for catching yet another mistake. We apologize for our carelessness. Yes, we only included 3 GMO risk items in the study. We excluded one of the four items which did not refer to an actual genetic risk, but a risk of big corporations having too much power, as this seemed to us to bring up a rather unrelated kind of concern of the business regarding GMO products. We have added a footnote in the manuscript now which says “The Scott et al. paper included an additional item about GMO risks that read ‘Genetically modified crops giving big corporations too much power over small farmers.’ We omitted this item from our study as it did not seem to tap into fears of genetic modifications per se, but concerns about implications of business practices regarding GMO products.”

Relatedly, the original endpoints in the Table 2 can be confusing. It makes it look like GMO attitudes is a measure where higher scores = more negative attitudes, but you’ve reverse coded it.
Why not rename the GMO attitudes measure so it’s clear what direction it goes in throughout the manuscript (for example: “Positive Attitudes to GMOs” or perhaps “GMO Acceptance”, as you do with your “eugenics acceptance” measure)?

We thank the Reviewer for this excellent point and we have changed the paper accordingly. We now refer to the GMO measure as “GMO Acceptance,” both in the Table, and in the Results section. Also, in Table 2 we have removed the labels for the original endpoints as those only refer to the risk items, and given that the entire scale is reverse-scored, we agree with the reviewer that this only increases confusion.

A number of items from my last review that have not been fully addressed.

1. (Essentialism Items as Capturing More than Just Essentialist Beliefs from last round): I cannot see the tables in your letter, which I assume is an issue with copying and pasting into the journal’s web interface. All I see is “Shorter BGD Vs original: ”. Can you please include those correlations in the next response letter?

There must have been an issue with how the correlations showed up in the letter on the online portal as the two tables of the correlations were indeed there on the original letter that we submitted. We include the two tables again here. In case it remains invisible to the Reviewer again we note that the shorter BGD measure correlated .233 with eugenic acceptance, .589 with the GET, .122 with SDO, and .118 with age – all of these are significant at p < .001, as they were with the original scale. Gender did not correlate with either BGD scale (-.007 with the shorter measure, and -.044 with the longer one). The alpha of the shorter scale is .85 in comparison to .86 for the entire scale.

Shorter BGD

Vs original:

2. (GMO-Essentialism Correlation point last round): I think the correlational nature and the issue of third variables of trust in science/knowledge should be more fully acknowledged in the limitations section than just a sentence or two. When I see the following findings--people who support eugenics are more negative to GMOs and that people who have more simplistic/deterministic views of genetics causes (essentialism) are more negative to GMOs--the first thing I wonder is whether this is all driven by some type of general trust in science or broad science education. This needs to be addressed in the manuscript.

We agree with the Reviewer about the limits of the correlational data, and we have added the following sentence to the Limitations Section on page 17. “In particular, it is possible that a third variable, such as general trust in science, cognitive style, or broad science education, may underlie the correlations of some of these variables, including attitudes towards GMOs, eugenics acceptance, and genetics knowledge.”

3. (#4 in Other Comments): Can you move the clarification you added in the study 1 results section up to the study 1 methods section about BGD and GET being two measures capturing the same thing? I still think it is confusing when you read the BGD and GET paragraphs.

We have now moved the sentence to the method section on page 11 which specifies “We included two distinct measures of genetic essentialist biases as a test of robustness.”

4. Variability in Correlations Across Studies. I think it matters that your effect sizes change a LOT in a near-direct replication. This should be acknowledged in the manuscript. One possibility is TurkPrime recruits more attentive MTurk workers, which reduces noise, but this probably doesn’t account for the entire difference.

We have now added a sentence on page 15 to acknowledge the difference in the magnitude in the correlations between the two studies, which reads: “Unexpectedly, these correlations were considerably larger than they were in Study 1, and we are not sure what aspect of the sample or survey design might be responsible for these differences in magnitude.”

Other: (page numbers from track changes version):
p. 2 “highlighting the value of education in genetics” doesn’t this phrase really go beyond the scope of your data, and imply a causal relationship?

We agree that this sentence implies a causal relationship, which to us seems like the most reasonable take on the underlying nature of the correlation, although we acknowledge that third variables very well may be responsible. We have slightly toned this down by adding the word “potentially” before highlighting.

p. 11 “24 item” should be 24-item; sclae should be scale in genetic essentialist tendencies too

p. 14 7 point should be 7-point

We have made these minor corrections now.

We thank the reviewers for their helpful comments and their kind words about our research.

On Behalf of the Authors,

Steve Heine
